# Structural and Functional Alterations Caused by Aureobasidin A in Clinical Resistant Strains of *Candida* spp.

**DOI:** 10.3390/jof9111115

**Published:** 2023-11-17

**Authors:** Rodrigo Rollin-Pinheiro, Daniel Clemente de Moraes, Brayan Bayona-Pacheco, Jose Alexandre da Rocha Curvelo, Giulia Maria Pires dos Santos-Freitas, Mariana Ingrid Dutra da Silva Xisto, Luana Pereira Borba-Santos, Sonia Rozental, Antonio Ferreira-Pereira, Eliana Barreto-Bergter

**Affiliations:** 1 Laboratório de Química Biológica de Microrganismos, Departamento de Microbiologia Geral, Instituto de Microbiologia Paulo de Góes, Universidade Federal do Rio de Janeiro (UFRJ), Rio de Janeiro 21941-902, Brazil; giuliapires89@gmail.com (G.M.P.d.S.-F.); marylanax@gmail.com (M.I.D.d.S.X.); 2 Laboratório de Bioquímica Microbiana, Departamento de Microbiologia Geral, Instituto de Microbiologia Paulo de Góes, Universidade Federal do Rio de Janeiro (UFRJ), Rio de Janeiro 21941-902, Brazil; danielcmoraes@micro.ufrj.br (D.C.d.M.); bbayona@uninorte.edu.co (B.B.-P.); alexandrecurvelo@hotmail.com (J.A.d.R.C.); apereira@micro.ufrj.br (A.F.-P.); 3 Departamento de Medicina, División Ciencias de la Salud, Universidad del Norte, Km 5, Vía Puerto Colombia, Área Metropolitana de Barranquilla, Barranquilla 081007, Colombia; 4 Programa de Biologia Celular e Parasitologia, Instituto de Biofísica Carlos Chagas Filho, Universidade Federal do Rio de Janeiro (UFRJ), Rio de Janeiro 21941-902, Brazil; luanaborba@biof.ufrj.br (L.P.B.-S.); rozental@biof.ufrj.br (S.R.)

**Keywords:** aureobasidin A, sphingolipids, *Candida*, membrane, fungal infection

## Abstract

*Candida* species are one of the most concerning causative agents of fungal infections in humans. The treatment of invasive *Candida* infections is based on the use of fluconazole, but the emergence of resistant isolates has been an increasing concern which has led to the study of alternative drugs with antifungal activity. Sphingolipids have been considered a promising target due to their roles in fungal growth and virulence. Inhibitors of the sphingolipid biosynthetic pathway have been described to display antifungal properties, such as myriocin and aureobasidin A, which are active against resistant *Candida* isolates. In the present study, aureobasidin A did not display antibiofilm activity nor synergism with amphotericin B, but its combination with fluconazole was effective against *Candida* biofilms and protected the host in an in vivo infection model. Alterations in treated cells revealed increased oxidative stress, reduced mitochondrial membrane potential and chitin content, as well as altered morphology, enhanced DNA leakage and a greater susceptibility to sodium dodecyl sulphate (SDS). In addition, it seems to inhibit the efflux pump CaCdr2p. All these data contribute to elucidating the role of aureobasidin A on fungal cells, especially evidencing its promising use in clinical resistant isolates of *Candida* species.

## 1. Introduction

*Candida* species are one of the most concerning causative agents of fungal infections in humans. They are responsible for a wide spectrum of diseases, which range from superficial mycosis to life-threatening disseminated candidiasis [1]. In the context of invasive infections, the most frequent species are *Candida albicans* and *Nakaseomyces glabrata* (formerly named *Candida glabrata*) [2]. The mortality rates of invasive candidiasis can reach up to 60%, especially in patients presenting predisposing conditions, such as HIV infection, organ transplantation, cancer and diabetes [3]. In healthcare environments, *Candida* infections are the most common fungal disease associated with nosocomial acquired infections [4].

The treatment of invasive *Candida* infections was based on the use of fluconazole, an azole antifungal drug that blocks the enzyme lanosterol 14-α demethylase and, thus, disrupts ergosterol synthesis [5,6]. However, the emergence of resistant isolates has been an increasing concern, especially those presenting multi-drug resistance based on the expression of different types of transporters of over-expressing azole targets [7]. For this reason, recommendations for candidiasis treatment have been recently changed and echinocandins are now considered the first choice of drug, followed by azoles [8,9,10,11]. Alternative antifungal therapy is limited due to a variety of factors, which include the absence of a wide diversity of antifungal classes and high levels of toxicity and side effects presented by the antifungal drugs currently available in clinical settings, which lead to the need for studies of new antifungal approaches [12].

Besides the class of azoles, there are only three other groups of antifungal molecules available in healthcare units, which target only three fungal cell structures: polyenes, which directly bind to ergosterol found in the plasma membrane and cause the release of cytoplasmic content; echinocandins, which inhibit the enzyme β(1,3)-glucan synthase and affect cell wall synthesis; and fluoropyrimidine analogs, which block DNA synthesis [13]. For these reasons, the development of new antifungal options, as well as the discovery of alternative targets in fungal cells, is an urgent need. In this context, sphingolipids have been considered a promising candidate as a new target on fungal cells due to its role on fungal growth, virulence and cellular signaling [14,15]. Recently, the group of molecules acylhydrazones has been shown to block glucosylceramide synthesis in different fungal pathogens, such as *C. neoformans*, *C. albicans*, *A. fumigatus* and *Pneumocystis murina*, but does not affect the lipid metabolism in mammalian cells [16]. Inhibitors of sphingolipid biosynthesis have already been described, such as myriocin and aureobasidin A, which presented antifungal activity against a variety of pathogenic fungi, such as *Aspergillus* and *Candida* species including *Candida auris* [17,18,19,20,21]. Aureobasidin A acts by blocking the inositol phosphoryl ceramide (IPC) synthase, which mediates the synthesis of IPCs that are one of the most important sphingolipids produced by fungi [22]. However, the sphingolipid biosynthesis is a complex metabolic pathway that can be modulated by fungal cells, as has already been demonstrated by lipidomic analysis in *Candida* species [21]. In addition, the use of sphingolipid inhibitors in pathogenic filamentous fungi, such as *Scedosporium* species, has revealed that many intermediate molecules from the biosynthetic pathway are affected when cells are treated with different inhibitors [23].

More recently, our group evidenced the antifungal effect of these inhibitors in fluconazole-resistant isolates of *C. albicans* and *C. glabrata* and their synergistic effect with fluconazole [24]. For these reasons, it is important to understand which modifications occur in *Candida* cells when treated with aureobasidin A. The present study aimed to elucidate the effect of aureobasidin A on fluconazole-resistant clinical strains of *C. albicans* and *C. glabrata* that overexpress MDR1, CDR1 and ERG11, which are typical mechanisms of resistance for azole antifungals, focusing its effect on fungal lipids and the interaction with antifungal drugs that target an important fungal lipid, ergosterol, such as fluconazole and amphotericin B.

## 2. Materials and Methods

### 2.1. Cell Lineages and Reagents

A total of five *Candida* strains were used in this study. *C. albicans* ATCC 10231D-5 and *C. glabrata* ATCC 2001D-5 were used as standard (ATTCC^©^, Manassas, VA, USA). Three clinical isolates displaying resistance patterns to fluconazole were also evaluated, *C. glabrata* 109 strain, *C. albicans* 1114 strain (both from our laboratory collection) and *C. albicans* 12-99 strain, kindly provided by Theodore White from University of Missouri, USA. For all experiments, the strains were grown on Yeast Extract Peptone Dextrose (YPD) agar and transferred to YPD broth and incubated at 37 °C for 18 h under agitation.

*Saccharomyces cerevisiae* mutant strains were also used: AD/1234567, deleted from all genes that encode ABC transporters related to the MDR phenotype (Pdr5p, Yor1p, Snq2p, Pdr10p, Pdr11p and Ycf1p); CaCDR1p+, CaCDR2p+, CaMDR1p+ and CgCDR1p+, that overexpress CaCdr1p, CaCdr2p, CaMdr1p and CgCdr1p, respectively, and present the same gene deletions as AD/1234567. Those mutants were kindly provided by Dr. Richard Cannon and Dr. Brian Monk (University of Otago—New Zealand).

Aureobasidin A (Sigma-Aldrich, St. Louis, MO, USA) and fluconazole (University Pharmacy, UFJF, Juiz de Fora-MG, Brazil) were used in susceptibility and synergism tests.

### 2.2. Susceptibility of Forming and Preformed Biofilms to Aureobasidin A and Fluconazole

Biofilm formation was analyzed according to Hacioglu and colleagues [25]. Firstly, an adhesion step was performed by adding 200 μL from a standardized suspension of *Candida* cells (1 × 10^7^/mL) to each well of a polystyrene microplate and incubated for 1.5 h at 37 °C. Then, the supernatant containing non-adherent cells was removed and RPMI 1640 medium supplemented with MOPS, 2% glucose and 20% fetal bovine serum (FBS, Gibco, Waltham, MA, USA) was added in the absence (positive control) or presence of aureobasidin A (4–0.125 μg/mL) and/or fluconazole (256–258 μg/mL). Adherent cells were then incubated for 48 h at 37 °C.

For the preformed biofilm assay, cells were grown as described above in the absence of the compounds. After 24 h of biofilm formation, the supernatant was removed and supplemented RPMI was added in the absence (positive control) or presence of aureobasidin A (4–0.125 μg/mL) and/or fluconazole (256–258 μg/mL). An additional incubation of 24 h at 37 °C was performed to evaluate the anti-biofilm activity.

Evaluation of both biofilm formation and preformed biofilms was carried out using three chromogenic assays as previously described [26,27]: crystal violet, safranin and XTT assays were used to analyze the overall biomass, extracellular matrix and metabolic activity, respectively.

### 2.3. Interaction of Aureobasidin A with Amphotericin B and Fluconazole

Interaction analysis of aureobasidin A with antifungals was performed using the combination with fluconazole against preformed biofilms and with amphotericin B against planktonic cells. Both experiments were performed using the checkerboard method according to Reis de Sá and colleagues [28]. For the analysis against preformed biofilms, after 24 h of biofilm formation in the absence of the compounds, the supernatant was removed and supplemented RPMI was added in the absence (positive control) or presence of aureobasidin A (4–0.125 μg/mL) and/or fluconazole (256–258 μg/mL) in different directions. An additional incubation of 24 h at 37 °C was performed to evaluate the anti-biofilm activity of different combinations of aureobasidin A and fluconazole. For the combination of aureobasidin A and amphotericin B against planktonic cells, *Candida* cells were treated with both drugs using the checkerboard method and the results were evaluated after a 48 h incubation at 37 °C.

The fractional inhibitory index (FICI) was calculated according to the formula (MIC combined/MIC drug A alone) + (MIC combined/MIC drug B alone), where A is aureobasidin A and drug B is fluconazole. Interaction was classified according to the following parameter: ≤0.5, synergistic interaction; >0.5 to ≤4, no interaction; >4, antagonistic effect [29].

Bliss independence model was performed according to Meletiadis and colleagues and Zhao and colleagues [30,31]. The following formula was used to assess the drug interaction: Eexp = Ea + Eb − Ea × Eb, in which Eexp is the expected efficacy of drug combination, Ea is the efficacy of drug A (aureobasidin A) and Eb is the efficacy of drug B (fluconazole). The results were classified as: synergistic effect, Eobs > Eexp; indifference, Eobs = Eexp; antagonistic effect, Eobs < Eexp.

### 2.4. Analyses of Cellular Alterations

Alterations of *Candida* cells caused by aureobasidin A were analyzed using fluorescent staining. Oxidative stress, mitochondrial membrane potential and the content of chitin and neutral lipids were evaluated using 2′,7′–dichlorofluorescein diacetate (DCFH-DA) (Sigma-Aldrich, St. Louis, MO, USA), JC-1 probe (ThermoFisher, Waltham, MA, USA), calcofluor white (Sigma-Aldrich, St. Louis, MO, USA) and Nile Red (Sigma-Aldrich, St. Louis, MO, USA), respectively. Cells were grown in the absence (positive control) or in the presence of 0.5× MIC of aureobasidin A for 48 h at 37 °C. Then, cells were stained for 1 h at 37 °C under protection from light with 50 μg/mL of DCFH-DA, 10 μg/mL of JC-1, 25 μg/mL of calcofluor white or 2 mg/mL of Nile Red [32,33]. Samples were washed three times to remove residual dye and suspended in PBS. Fluorescence intensity was measured using the SpectraMax 340 microplate reader (Molecular Devices, San Jose, CA, USA) at the following conditions: DCFH-DA at 492 nm (excitation) and 517 nm (emission); JC-1 at 475 nm (excitation) and 529 nm (green fluorescence) or 590 nm (red fluorescence) for the calculation of the red/green fluorescence intensity; calcofluor white at 350 nm (excitation) and 432 nm (emission); Nile Red at 550 nm (excitation) and 635 nm (emission).

### 2.5. Susceptibility to SDS

The susceptibility to sodium dodecyl sulphate (SDS) was analyzed using 1 × 10^5^ cells grown in 96-well plates containing supplemented RPMI in the absence (positive control) and in the presence of sub-inhibitory concentrations of aureobasidin A (0.5× MIC) for 24 h at 37 °C [23]. Then, the supernatant was removed from the microplates and SDS (90 μg/mL) was added. After another 24 h incubation, the cell viability was measured using an XTT reduction assay and readings were captured using a spectrophotometer (Bio-Rad, Hercules, CA, USA) at 490 nm.

### 2.6. Evaluation of Cell Membrane Permeability

The cell membrane permeability was determined by measuring the release of DNA to the culture supernatant [34]. *Candida* cells (1 × 10^5^) were grown in supplemented RPMI in the absence (positive control) or presence of aureobasidin A (0.5× MIC) for 48 h at 37 °C. After the incubation time, the cells were pelleted and the supernatant analyzed using a NanoDrop 2000 spectrophotometer (Thermo Fisher Scientific) to quantify free DNA (260 nm). A sterile culture medium sample was used as a negative control.

### 2.7. Efflux Pump Inhibition

The ability of aureobasidin A in inhibiting efflux pumps was evaluated through checkerboard assays. Briefly, *S. cerevisiae* cells (2 × 10^4^/mL) were incubated in YPD broth in the presence of combinations of 2-fold serial dilutions of aureobasidin A (1.25–0.078 μg/mL) and fluconazole (500–1.95 μg/mL) at 30 °C for 48 h. Cell growth was measured using a 600 nm SpectraMax 340 microplate reader (Molecular Devices, San Jose, CA, USA), and interactions were assessed through a FICI model, as described in Section 2.3.

### 2.8. Scanning Electron Microscopy

Scanning electron microscopy was performed according to Rollin-Pinheiro et al. [35]. *Candida* cells were grown in supplemented RPMI in the absence (positive control) or in the presence of 0.5× MIC of aureobasidin A and incubated for 48 h at 37 °C. After the period of incubation, the cell growth was collected and processed for scanning electron microscopy. Briefly, samples were processed as follows: i. fixation in 2.5% glutaraldehyde and 4% formaldehyde, in 0.1 M cacodylate buffer, for 30 min at room temperature; ii. post-fixation in 1% osmium tetroxide in 0.1 M cacodylate buffer containing 1.25% potassium ferrocyanide for 30 min; iii. dehydration in a graded ethanol series (30–100%); iv. critical point drying in CO_2_ (EM CPD300, Leica, German); v. adhesion to aluminum stubs with carbon tape; and vi. coating with gold.

Images were obtained with FEI Quanta 250 scanning electron microscope (FEI Company, Hillsboro, OR, USA) and processed using Photoshop v.CS6 software (Adobe, San Jose, CA, USA). The yeast area was measured using the Image J v.1.52a software.

### 2.9. Caenorhabidtis Elegans Infection Assay

The in vivo infection assay used AU-37 *C. elegans* strain that was acquired by the *Caenorhabidtis* Genetics Center (CGC), University of Minnesota (USA). The worms were maintained in nematoid growth agar medium (NGM) (3 g/L of NaCl; 17 g/L of agar; 2.5 g/L of peptone; 1 mM CaCl_2_; 5 mg/L of cholesterol in ethanol; 1 mM MgSO_4_ and 25 mM KPO_4_) at 15 °C, seeded with the strain of *Escherichia coli* OP50 that was used as the regular food source for these nematodes.

To assess the influence of 0.25 µg/mL aureobasidin A associated or not with 128 µg/mL of fluconazole on nematode infection caused by the three strains independently, Priya’s [36] methodology was used with minor modifications, as follows. A synchronized population of nematodes was obtained by collecting eggs from pregnant adult larvae with M9 buffer (3 g of KH_2_PO_4_, 6 g of Na_2_HPO_4_, 5 g of NaCl, 1 mL of 1 M MgSO_4_ in 1 L of H_2_O) and vigorously vortexed with 0.5 mL of 5 M NaOH and 1.5 mL of 6% sodium hypochlorite in 35 s sequential cycles. Released eggs were then centrifuged at 1300× *g* for 50 s, cleaned in sequential washes in M9, added to plates containing NGM medium seeded with *E. coli* OP50 and incubated for 72 h at 25 °C to obtain synchronized larvae in stage L4 [37]. Infection of synchronized larvae with *C. albicans* 12-99, *C. albicans* 1114 and *C. glabrata* 109 was carried out as proposed by Tempakakis and colleagues [38], with minor modifications. Briefly, 5 × 10^5^ cells of each strain were incubated in Petri dishes containing BHI agar supplemented with 90 µg/mL of kanamycin, 200 µg/mL of streptomycin and 200 µg/mL of ampicillin for 24 h at 37 °C. The plates were cooled to at room temperature and approximately 100 synchronized worms in stage L4 were added onto each plate seeded with different *Candida* strains and allowed to feed for 180 min at 25 °C and allowed to crawl on agar surface with the aim of feeding on yeast. Infected worms were then washed serially in M9 buffer and placed on clean BHI plates to crawl for 40 min, aiming to remove yeast from their cuticles [39]. Nematodes were collected from plates and resuspended in screen medium (60% M9, 40% BHI, 90 µg/mL kanamycin, 200 µg/mL streptomycin and 200 µg/mL ampicillin). To evaluate the influence of aureobasidin associated or not with fluconazole on nematode infection, 20 infected worms were incubated in 96-well TPP^®^ plates (Schaffhausen, Switzerland) containing screen medium with 0.25 µg/mL aureobasidin alone or associated with 128 µg/mL of fluconazole and incubated at 25 °C for nine days maximum without shaking and scored as live or dead on daily basis. The survival of nematodes was monitored in an inverted microscope Axiovert100 Zeiss^®^ (Jena, Germany) and determined through motility, sinus morphology and/or bulbopharyngeal movement of each helminth. The survival index was calculated from the proportion of the viable population in relation to the total number of worms, including live and dead animals.

### 2.10. Statistical Analyses

All experiments were performed in triplicate in three independent experimental sets. Statistical analyses were performed using GraphPad Prism v5.00 for Windows (GraphPad Software, San Diego, CA, USA). The nonparametric Kruskal–Wallis one-way analysis of variance was used to compare the observed differences among the groups, and individual comparisons of the groups were performed using a Bonferroni post-test. The 90% or 95% confidence interval was determined in all experiments.

For the in vivo analysis, the experiment was conducted independently two-fold, with a quadruplicate analysis in each one. Data were analyzed by Student’s paired *t*-test, and *p* values lower than 0.05 were considered significant.

## 3. Results

### 3.1. Interaction between Aureobasidin A with Amphotericin B

In a previous study from our group it has been demonstrated that aureobasidin A displays synergistic effects with fluconazole in planktonic cells. For this reason, it was evaluated whether it also has synergism with amphotericin B, another antifungal agent used in clinical settings for the treatment of candidiasis. For the three *Candida* strains used, the interaction between aureobasidin A and amphotericin B was indifferent, indicating that there was no synergistic or antagonistic effect between the compounds (Table 1).

### 3.2. Effect of Aureobasidin A against Candida Biofilms

In previous studies from our group, it has been demonstrated that aureobasidin A is active against *Candida* planktonic cells, including clinical resistant isolates (109, 1114 and 12-99). *Candida glabrata* 109 and *Candida albicans* 1114 overexpress the CDR1 (ATP transporter) and MDR1 (MFS transporter) genes, respectively, whereas *Candida albicans* 12-99 overexpress the ERG11, CDR1, CDR2 and MDR1 genes [24].

**Figure 1:**
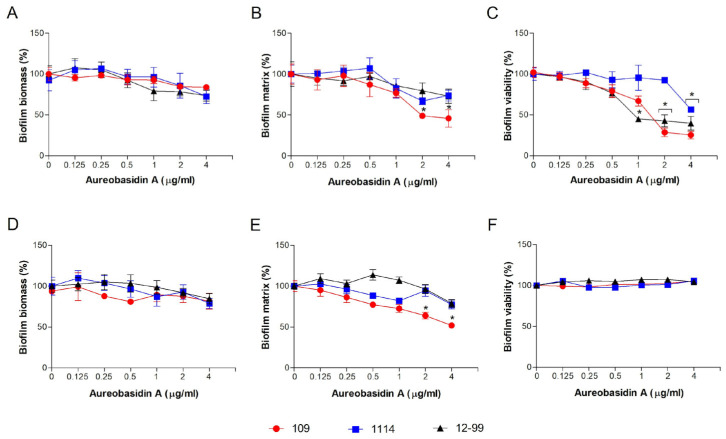
Effect of aureobasidin A on forming (**A**–**C**) and preformed (**D**–**F**) *Candida* biofilms. 109: *C. glabrata* 109; 1114: *C. albicans* 1114; 12-99: *C. albicans* 12-99. Fungal biomass (**A**,**D**), extracellular matrix (**B**,**E**) and viability (**C**,**F**) were measured using violet crystal, safranin and XTT reduction assay, respectively. * *p* < 0.05.

The present study aimed to investigate the effect of aureobasidin A against biofilms produced by these three strains. The evaluation of forming biofilm revealed that it did not affect fungal biomass (Figure 1A) but reduced approximately 50% of the extracellular matrix at 2 and 4 μg/mL in strain 109 (Figure 1B). A decrease of at least 50% of biofilm viability was also observed in strain 109 at 2 and 4 μg/mL and in the strain 12-99 at 1, 2 and 4 μg/mL (Figure 1C). In strain 1114, aureobasidin A reduced biofilm viability at 4 μg/mL to 60% compared to the control (Figure 1C).

Regarding the preformed biofilm, when biofilm was formed prior to the treatment with aureobasidin A, it did not affect fungal biomass or viability in any *Candida* isolates (Figure 1D,F), but a reduction in the extracellular matrix was observed in strain 109 at 2 and 4 μg/mL (Figure 1E).

### 3.3. Effect of Aureobasidin A Combined with Fluconazole against Candida Biofilms

Considering that the interaction between aureobasidin A and fluconazole displayed synergism in planktonic cells, as demonstrated in previous a study [24], it was analyzed whether the same effect is observed when *Candida* cells are grown as biofilms. Using the FIC index method, there was no synergistic effect between aureobasidin A and fluconazole for any of the three *Candida* isolates (Table 2). However, when the data were analyzed by the BLISS method, a synergistic effect was observed in the strains 1114 and 12-99 (Table 3).

### 3.4. Alterations of Cellular Parameters Caused by Aureobasidin A

**Figure 2:**
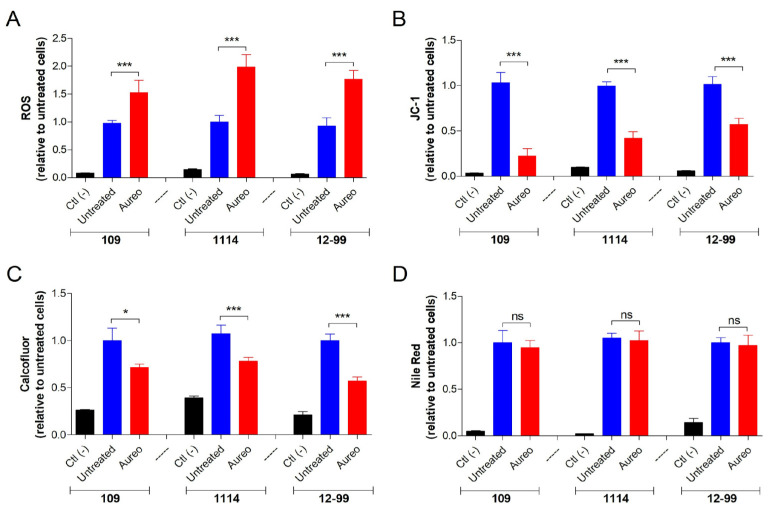
Cellular alterations caused by aureobasidin A on *C. glabrata* 109, *C. albicans* 1114 and *C. albicans* 12-99 after incubation with the compound for 48 h at 37 °C. Oxidative stress, mitochondrial membrane potential, chitin and neutral lipid contents were measured using the fluorescent probes DCFDA, JC-1, calcofluor white and Nile Red, respectively. * *p* < 0.05; *** *p* < 0.001. ns = non significative.

Since aureobasidin A displayed significant antifungal activity, it was investigated which alterations are observed in treated *Candida* cells. DCFDA, JC-1, calcofluor white and Nile Red were used to evaluate the oxidative stress, depolarization of mitochondrial membrane, chitin and neutral lipids content, respectively. Treatment with aureobasidin A led to an increase in ROS production in both the 109, 1114 and 12-99 strains between 1.5 and 2.0 fold compared to the control, suggesting that the compound resulted in oxidative stress (Figure 2A). JC-1 staining was at least 50% decreased in the treated cells, indicating that aureobasidin A caused depolarization of the mitochondrial membrane (Figure 2B). Regarding the analyses of molecules from the fungal surface, the chitin content measured by calcofluor white was around 30–40% reduced by aureobasidin A treatment (Figure 2C), whereas the staining of neutral lipids by Nile Red was not altered in the treated cells (Figure 2D).

### 3.5. Scanning Electron Microscopy

**Figure 3:**
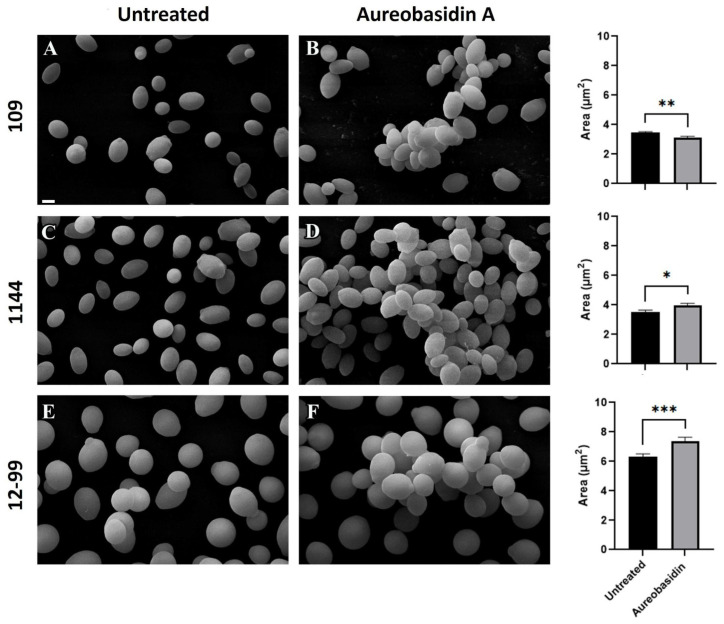
Scanning electron microscopy of *C. glabrata* 109, *C. albicans* 1114 and *C. albicans* 12-99 grown for 48 h at 37 °C in the presence or the absence of aureobasidin A. Cell area was measured using the software Image J v.1.52a. Untreated (**A**,**C**,**E**): strains 109, 1114 and 12-99, respectively, grown in the absence of aureobasidin A. Aureobasidin A (**B**,**D**,**F**): strains 109, 1114 and 12-99, respectively, grown in the presence of the compound. * *p* < 0.05; ** *p* < 0.01; *** *p* < 0.001.

Since the treatment of *Candida* cells with aurebasidin A revealed some cellular alterations, modifications on fungal morphology were evaluated by scanning electron microscopy. Compared to the control of untreated cells, which showed isolated yeast cells, all the three strains presented grouped cells when incubated with aureobasidin A (Figure 3). In addition, the area of yeast cells was measured and it revealed that the cell size of *C. glabrata* 109 was reduced when treated with aurebasidin A, whereas it was increased in *C. albicans* 1114 and 12-99 (Figure 3).

### 3.6. Susceptibility to SDS in the Presence of Aureobasidin A

**Figure 4:**
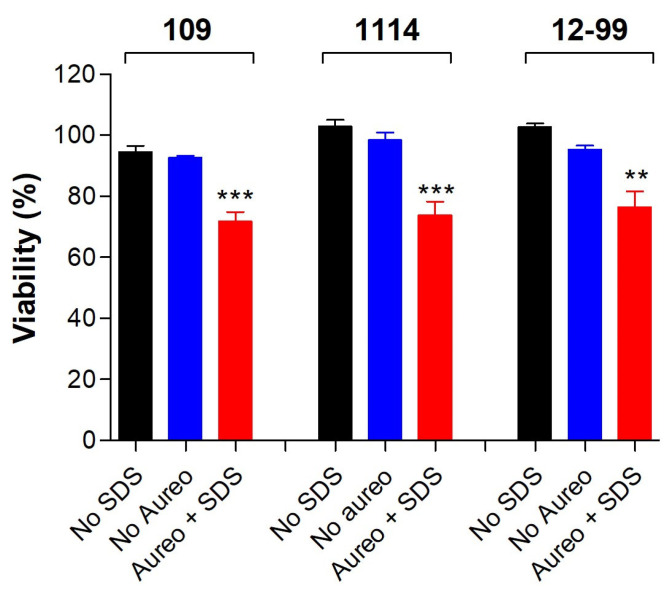
Susceptibility of *C. glabrata* 109, *C. albicans* 1114 and *C. albicans* 12-99 to aureobasidin A in the presence of the membrane stressor SDS. Cells were grown for 48 h at 37 °C in the absence or the presence of ½ MIC of aureobasidin A and a subinhibitory concentration of SDS. Cell viability was assessed using the XTT reduction assay. No SDS: cells grown in the absence of both SDS and aureobasidin A. No Aureo: cells grown in the presence of SDS and the absence of aureobasidin A. Aureo + SDS: cells grown in the presence of both SDS and aureobasidin A. ** *p* < 0.01; *** *p* < 0.001.

Considering that aureobasidin A inhibits the synthesis of an important class of lipids found on fungal membrane, sodium dodecyl sulphate (SDS) was used as a stressor of plasma membrane to check whether the compound could increase the susceptibility of *Candida* cells to SDS. To do this, a subinhibitory concentration of SDS was used to check whether the treatment with aureobasidin A increased the susceptibility of *Candida* cells to the stressor. The viability of all the three strains was reduced when treated with ½ MIC of aureobasidin A, suggesting that it enhances the susceptibility of fungal cells to SDS (Figure 4).

### 3.7. Membrane Permeability in the Presence of Aureobasidin A

**Figure 5:**
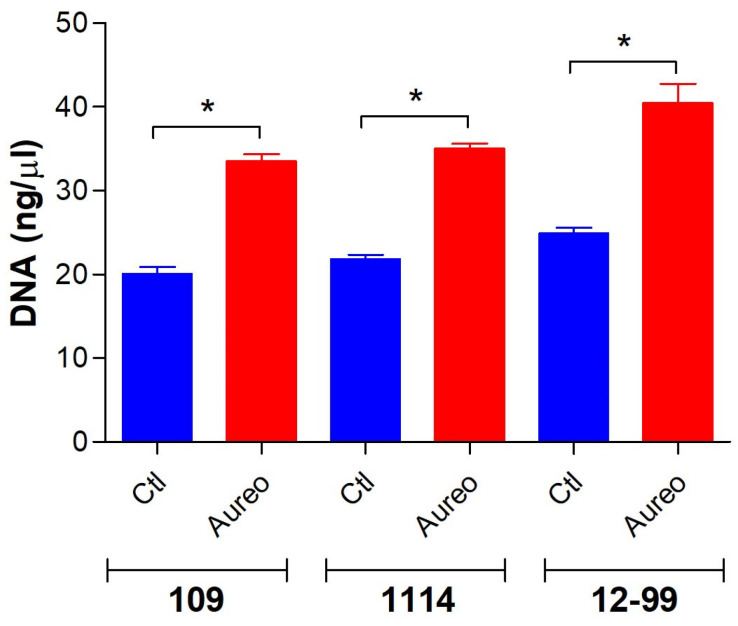
Analysis of DNA content in the supernatant of fungal growth. *C. glabrata* 109, *C. albicans* 1114 and *C. albicans* 12-99 were grown for 48 h at 37 °C in the absence (CTL) or the presence (AUREO) of aureobasidin A. After centrifugation, supernatant was collected and DNA content was measured by NanoDrop 2000 spectrophotometer at 260 nm. * *p* < 0.001.

Considering that the treatment with aureobasidin A resulted in more susceptible cells, the permeability of fungal membrane in the presence of the compound was investigated by measuring the DNA concentration found in the supernatant of culture medium. Whereas the control cells displayed around 20 ng/μL of DNA, the treatment with aureobasidin A resulted in an increase in DNA content in the supernatant of 30–40 ng/μL (Figure 5), suggesting that the compound could induce the permeability of fungal membranes.

### 3.8. Efflux Pump Inhibition

Checkerboard assays performed with *S. cerevisiae* mutant strains show that aureobasidin A displays antifungal activity against *S. cerevisiae*, with MIC of 1.25 μg/mL for the five strains tested. Fluconazole presented 0.5 μg/mL MIC against AD/1234567, 125 μg/mL against CaCDR2p+ and CaMDR1p+ strains, and 500 μg/mL against CaCDR1p+ and CgCDR1p+ strains. Combining aureobasidin A with fluconazole led to FICI values of 0.52–2.00 against the AD/1234567, CaCDR1p+, CaMDR1p+ and CgCDR1p+ strains, indicating indifferent interactions. The association between the compounds against CaCDR2p+ resulted in a synergistic interaction, with a FICI value of 0.28 (Table 4).

### 3.9. In Vivo Assay

Based on previous results that evaluated if aureobasidin A was synergic in vitro with fluconazole, enhancing its effect on different resistant *Candida* strains with specific resistant patterns [24], aureobasidin A was also tested in vivo in a *C. elegans* model of *Candida* infection to evaluate the behavior of the aureobasidin A/fluconazole interaction as a treatment to counteract *Candida* infection in the immunocompromised *C. elegans* AU-37. Briefly, worms were infected with each *Candida* strain and then treated with 0.25 µg/mL of aureobasidin A, associated or not with 128 µg/mL fluconazole.

**Figure 6:**
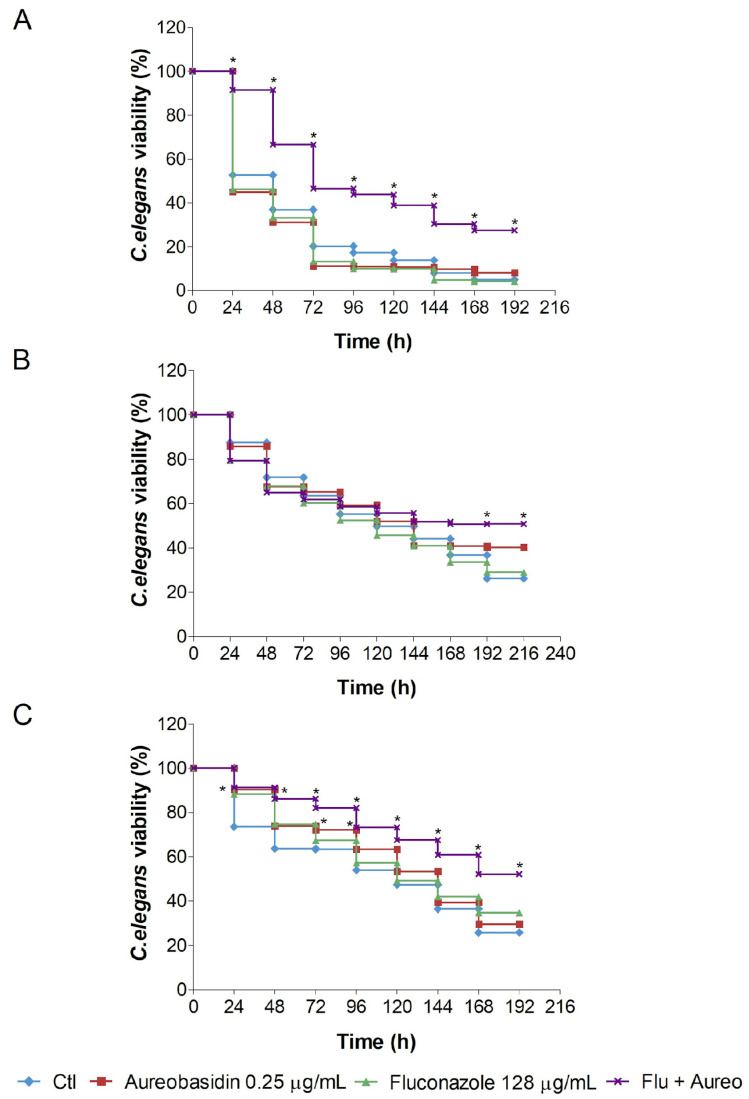
In vivo assay using the *C. elegans* model of infection. After infection by *C. glabrata* 109 (**A**), *C. albicans* 1114 (**B**) or *C. albicans* 12-99 (**C**), worms were treated with aureobasidin A, fluconazole or the combination aureobasidin A/fluconazole (Flu + Aureo). CTL: infected worms grown with no treatment regimen. *C. elegans* viability was measured at every 24 h interval. * *p* < 0.001.

As presented in Figure 6, more than 47.3% of the worms infected with *C. glabrata* 109 died after one day and only 5% were alive after seven days. A very similar result was observed for worms treated with 128 µg/mL fluconazole, with more than 55% of worms dead one day after treatment and more than 90% of worms dead by the seventh day post-infection. Infected worms treated with 0.25 µg/mL of aureobasidin A exhibited a slight but non-significant prolonged survival. However, when worms were treated with fluconazole and aureobasidin A combined, infection was significantly reduced in all times tested. Nematodes survival reached 91.5, 66.6, 46.5, 43.8, 38.8, 30.4 and 27.5% for 24, 48, 72, 96, 12, 144 and 168 h post infection, respectively (Figure 6A).

The infection caused by the *C. albicans* 1114 strain demonstrated a controversial result that is represented in Figure 6B. Such infection evolved in a more stable manner but reached no significant efficacy in any treatment test until the fifth post-treatment day, where the survival rate significantly reached 51.7, 50.7 and 50.8% for combined treatment against 44.1, 36.8 and 26.2% for untreated infected nematodes on the fifth, sixth and seventh day, respectively (Figure 6B).

Lastly, the infection caused by the *C. albicans* 12-99 strain, although it evolved in a more stable manner as well, revealed a treatment efficacy resembling the one exposed by the infection caused by strain 109 (Figure 6C). The treatment with the association of aureobasidin A and fluconazole showed a significant survival rate compared with the untreated worms for all times tested. This rate was 91.2, 86.1, 82, 73.2, 67.5, 60.8, 52.1% against 73.5, 63.6, 63.3, 53.9, 47.3, 36.4 and 25.8% for the first to the seventh day, respectively. Nevertheless, the treatment with 0.25 µg/mL of aureobasidin A alone was also able to significantly enhance worms’ survival in the first four post-infection days compared with the untreated cells. This survival percentage was calculated as 90.4, 73.9, 72.1 and 63.3% for the first to the fourth day, respectively (Figure 6C).

## 4. Discussion

A recent study from our group has demonstrated that aureobasidin A is active against resistant clinical isolates of *Candida* species (109, 1114 and 12-99) and displays a synergistic effect with fluconazole [24]. Nevertheless, there are no studies showing what occurs with fungal cells when sphingolipid synthesis is impaired by inhibitors.

Since aureobasidin A is active against planktonic cells, as seen in the previous study, its effect on *Candida* biofilms was evaluated. Whereas it was active against biofilm formation, especially reducing the extracellular matrix and biofilm viability, a less prominent effect was observed against preformed biofilms. Tan and Tay [19] have also tested aurebasidin A against *Candida* biofilms and it has been shown that its susceptibility moved from 1 and 4 μg/mL in *C. glabrata* and *C. albicans* planktonic cells, respectively, to >64 μg/mL in biofilms of both species. Similar results have been observed for other *Candida* species [19]. These data indicate that fungal biofilms are more resistant to aureobasidin A, which has already been observed for many other antifungal molecules.

Considering that aureobasidin A and fluconazole presented synergistic effects [24], the interaction between aureobasidin A and amphotericin B was checked but no synergistic effect was observed. On the other hand, we also evaluated the combination of aureobasidin A and fluconazole against biofilms. Although no synergism was seen by the FIC index, BLISS method revealed a promising positive interaction between the drugs, especially for strain 12-99. Analyses of drug interaction involving the use of aureobasidin are scarce in the literature; therefore, more studies are needed to elucidate how this inhibitor interacts with the antifungal agents currently used in clinical settings.

Although it is well known that aureobasidin A acts on sphingolipid biosynthesis, there are few data in the literature showing modifications caused in fungal treated cells. Aureobasidin A MIC in *Cryptococcus neoformans* was not affected in the presence of sorbitol, suggesting that the compound does not interfere with the synthesis of cell wall components [40]. However, our data showed a decrease in chitin content measured using calcofluor white, suggesting that disruption of the sphingolipid biosynthetic pathway may result in modifications of the structure of the cell wall. On the other hand, it has been shown that the expression of the Cxt1p gene in *C. neoformans*, which is responsible for the synthesis of galactoxylomannan of the fungal capsule, is decreased when cells are treated with aureobasidin A [40]. It suggests that the biosynthesis of other polysaccharides could also be affected by the compound, which would corroborate the reduction in chitin content observed in *Candida* cells in our data; but, more studies are needed to elucidate the effect of aureobasidin A on cell wall components.

We found that *Candida* cells displayed increased oxidative stress and decreased mitochondrial membrane potential after aureobasidin A treatment, suggesting that the disruption of sphingolipid biosynthesis may also result in mitochondrial damage. In *C. neoformans*, it has also been shown that aureobasidin A treatment led to the production of oxidative stress, probably because of mitochondrial membrane disturbance and a reduction in lysosomal integrity [40]. It might indicate that the synthesis of sphingolipids is crucial for the integrity of fungal mitochondria.

Scanning electron microscopy revealed that treatment with aureobasidin A resulted in grouped cells compared to the control. Morphological alterations have also been demonstrated in *C. neoformans* by transmission electron microscopy, in which cells displayed excessive vacuolization and deposition of lipid globules [40].

Considering that aureobasidin A inhibits the synthesis of sphingolipids, which compose fungal membranes, it is reasonable to investigate in more detail the modifications it causes in *Candida* membrane integrity. Aureobasidin A increased the susceptibility of treated cells to SDS and induced the release of DNA to the supernatant, suggesting that sphingolipids might play a role in membrane integrity and susceptibility to detergents. Similar results were found for other eukaryotic cells, such as *Saccharomyces cerevisiae*, *C. neoformans* and *Toxoplasma gondii*, in which crushed vacuoles, leakage of ions K^+^ and the loss of intracellular structures, respectively, were observed [40,41,42]. All these data indicate that the inhibition of sphingolipid causes a variety of consequences on cellular membranes.

In *S. cerevisiae*, deletion of *ERG6* leads to resistance to aureobasidin A, indicating that alterations in genes responsible for ergosterol biosynthesis impact the susceptibility to sphingolipid inhibitors [43]. Interestingly, the *C. albicans* 12-99 strain, which overexpresses *ERG11*, is susceptible to aureobasidin A, suggesting that other genes from the ergosterol biosynthetic pathway are not related to the susceptibility to sphingolipid inhibitors. These data demonstrate that ergosterol and sphingolipid biosynthesis can be correlated.

Mishra and Prasad [44] have described the lipid distribution in *C. albicans* cells. Phospholipids and free fatty acids are the major lipids found in plasma membrane, followed by ergosterol and sphingolipids. Lipids are also present in the fungal cell wall, including ergosterol, fatty acid, phospholipids and sphingolipids, and its presence in the cell wall has been suggested to regulate *C. albicans’* adhesion to host cells [44]. These data could help to explain why cells treated with aurebasidin A seem to be linked to each other, as observed by scanning electron microscopy, but more studies are needed to elucidate it.

To evaluate whether aureobasidin A affects efflux pumps from *C. albicans* and *C. glabrata*, *S. cerevisiae* mutant strains that overexpress *Candida* MDR transporters were used. Since each *S. cerevisiae* strain harbors one specific efflux pump, it is possible to assess exactly which one is inhibited by a drug. Data showed that aureobasidin A inhibits CaCdr2p, due to the synergism observed. Moreover, combining aureobasidin A with fluconazole reduced the MIC of the antifungal by 64 times against CaMDR1p+, indicating that CaMdr1p is also inhibited by the same compound. It would explain the synergistic interactions between aureobasidin A and fluconazole against 1114, a *C. albicans* isolate that overexpresses CaMdr1p, and 12-99, a strain that overexpresses CaCdr1p, CaCdr2p and CaMdr1p [24]. Moreover, aureobasidin A displayed the same antifungal activity against all the *S. cerevisiae* strains tested in this study, indicating that this substance is not a substrate for the transporters. Therefore, it may be presumed that: (1) efflux inhibition by aureobasidin A is not related to competitive inhibition at the catalytic site of the transporters; and (2) *Candida* spp. would not become resistant to aureobasidin A over time. Corroborating our data, it has already been demonstrated that the transporter Cdr1p is localized in enriched ergosterol and sphingolipid microdomains [45], and the deletion of the *IPT1* (inositol phosphotransferase) gene decreases the level of Cdr1p on plasma membranes [46].

The in vivo efficacy of aureobasidin A was assessed using the *C. elegans* model of infection. For all of the three strains, the combination of aureobasidin A with fluconazole was effective in increasing the worm’s survival, corroborating the fact that aureobasidin A and fluconazole display in vitro synergism against *Candida* species. In vivo studies investigating the antifungal activity of aureobasidin A are rare in the literature. Takesako and colleagues [47] have performed a murine model of candidiasis and have demonstrated that the treatment with aureobasidin A protected mice against *Candida* infections. In addition, a previous study has shown that aureobasidin A is not toxic for *C. elegans* [24], indicating that its low toxicity is displayed not only in in vitro cell cultures, but also in in vivo analyses. These data suggest that it is a promising effective drug in the context of the disease established in a host.

All the data presented here bring a more detailed analysis of how aureobasidin A affects *Candida* cells, especially clinical resistant strains. Nevertheless, further studies are needed to test more *Candida* isolates and species, elucidate how sphingolipid biosynthesis is modulated by aureobasidin A and clarify how it interacts with antifungal agents. On the other hand, the evidence of an alternative drug with low MIC values, non-toxicity properties and in vivo efficacy to treat resistant isolates is a promising area to be explored to improve the management of non-susceptible invasive candidiasis in clinical settings.

## Figures and Tables

**Table 1:** Checkerboard assays of aureobasidin A combined with amphotericin B against *Candida* spp. clinical isolates.

Strains	Aureobasidin (µg/mL)			Amphotericin B (µg/mL)				
	MIC a	MIC c	FIC	MIC a	MIC c	FIC	FICI	Outcome
12-99	>0.25	>0.25	1	0.25	0.25	1	2	I
1114	>0.25	>0.25	1	0.25	0.25	1	2	I
109	>0.25	>0.25	1	0.25	0.25	1	2	I

MIC a, MIC of compound alone; MIC c, MIC of compound combined; FIC, fractional inhibitory concentration; FICI, fractional inhibitory concentration index; I, indifferent.

**Table 2:** Anti-biofilm activity of aureobasidin A and fluconazole—alone or in combination—using the Fractional Inhibitory Concentration Index (FICI) against *C. glabrata* 109, *C. albicans* 1114 and *C. albicans* 12-99.

Strains	Aureobasidin (µg/mL)			Fluconazole (µg/mL)				
	MIC a	MIC c	FIC	MIC a	MIC c	FIC	FICI	Outcome
12-99	>4	2	0.5	>256	64	0.25	0.75	I
1114	>4	2	0.5	>256	64	0.25	0.75	I
109	>4	2	0.5	>256	128	0.5	1.0	I

MIC a, MIC of compound alone; MIC c, MIC of compound combined; FIC, fractional inhibitory concentration; FICI, fractional inhibitory concentration index; I, indifferent.

**Table 3:** Anti-biofilm activity of aureobasidin A and fluconazole—alone or in combination—using the Bliss Independence method against *C. glabrata* 109, *C. albicans* 1114 and *C. albicans* 12-99.

		Efficacy of Combined Drugs			
Strains	Drugs	MIC_50_ (μg/mL)	*E*_obs_	*E*_exp_	Δ*E*, % (Interaction)
109	Fluconazole	>256	3.1	4.0	−0.9 (A)
	Aureobasidin A	>4.0			
1114	Fluconazole	>256	10.0	4.1	5.9 (S)
	Aureobasidin A	>4.0			
12-99	Fluconazole	>256	33.3	4.2	29.1 (S)
	Aureobasidin A	>2.0			

MIC, Minimal inhibitory concentration. E_obs_, efficacy observed in the analysis. E_exp_, efficacy expected according to Bliss calculation. ΔE, difference between E_obs_ and E_exp_.

**Table 4:** Checkerboard assays of aureobasidin A against *S. cerevisiae* mutant strains overexpressing *Candida* spp. efflux pumps.

Strains	Aureobasidin (µg/mL)			Fluconazole (µg/mL)				
	MIC a	MIC c	FIC	MIC a	MIC c	FIC	FICI	Outcome
AD/1234567	1.25	1.25	1.00	0.5	0.5	1.00	2.00	I
CaCDR1p+	1.25	0.63	0.50	500	125	0.25	0.75	I
CaCDR2p+	1.25	0.31	0.25	125	3.91	0.03	0.28	S
CaMDR1p+	1.25	0.63	0.50	125	2.00	0.02	0.52	I
CgCDR1p+	1.25	0.63	0.50	500	125	0.25	0.75	I

MIC a, MIC of compound alone; MIC c, MIC of compound combined; FIC, fractional inhibitory concentration; FICI, fractional inhibitory concentration index; I, indifferent; S, synergistic.

## Data Availability

Data are contained within the article.
